# Shockwave Intravascular Lithotripsy Use in the Femoro-Popliteal Segment: Considerations From an Expert Pan-European Panel Regarding Best-Care Practice

**DOI:** 10.1177/15266028241266417

**Published:** 2024-08-12

**Authors:** Athanasios Saratzis, Sarah Jane Messeder, Narayanan Thulasidasan

**Affiliations:** 1 Department of Cardiovascular Sciences, University of Leicester, Leicester, UK; 2 Department of Interventional Radiology, Guy’s and St Thomas’ NHS Foundation Trust, London, UK

**Keywords:** peripheral arterial disease, shockwave, intravascular lithotripsy, femoro-popliteal, vessel preparation, endovascular

## Abstract

**Purpose:**

Produce expert recommendations regarding the optimal use of Shockwave intravascular lithotripsy (IVL) when treating femoro-popliteal steno-occlusive peripheral artery disease (PAD), guiding operators to use Shockwave IVL.

**Materials and Methods:**

A modified 3-step Delphi process was used to gain consensus surrounding preoperative/intraoperative/postoperative considerations when using Shockwave IVL for femoro-popliteal PAD. This included a structured survey, focus-group (with qualitative thematic analysis of views expressed), and final confirmatory round; participants were recruited across Europe including the United Kingdom/Switzerland.

**Results:**

Following a review to inform an online survey, 25 experts took part in a survey (5 European countries, 2023), followed by a focus-group (15 participants), 9 interviews, and final confirmatory round. A list of recommendations was prepared where at least moderate-level or high-level agreement was reached (≥70% participants agreeing). The recommendations relate to the optimal preoperative imaging, preoperative preparation(s), intraoperative imaging and use of adjuncts, as well as postoperative course, when using Shockwave IVL.

**Conclusion:**

A list of expert recommendations is provided guiding the optimal use of Shockwave IVL in femoro-popliteal PAD. This will help operators achieve better clinical outcomes.

**Clinical Impact:**

This pan-European panel of experts using intravascular lithotripsy in routine peripheral arterial disease endovascular practice has provided important insights into best care practices before, during, and after such procedures. Several recommendations have been produced based on a structured consensus process to guide clinicians globally. This will improve and standardise the use of this technology in the femoro-popliteal arterial segment.

## Introduction

Peripheral artery disease (PAD) represents a major health problem worldwide; PAD affects a fifth of people over the age of 60 years in the United Kingdom and Europe. It is the commonest cause of lower limb amputations.

Most patients with chronic limb-threatening ischemia (CLTI) and some patients with severe claudication will require lower limb revascularization, one of the commonest vascular procedures in contemporary vascular practice, typically in the form of endovascular reconstruction. Arterial wall calcification represents a major challenge for endovascular arterial revascularization, often resulting in unsuccessful recanalization, dissection, or early restenosis. Shockwave intravascular lithotripsy (IVL) has emerged, as a vessel/lesion preparation strategy, to help overcome these issues.^1–4^

Efficacy-driven randomized evidence has shown that the use of Shockwave IVL in such lesions can reduce dissection rates and use of stenting; however, the specifics surrounding the use of this relatively new technology in order to achieve optimal outcomes have not been fully elucidated. The femoro-popliteal segment is the most common anatomical area affected by steno-occlusive disease and these lesions tend to be heavily calcified, especially in those with CLTI.^5–8^

In this process, the aim was to provide a best-practice guide regarding the use of Shockwave IVL preoperatively/intraoperatively/postoperatively, based on pan-European consensus within experts with considerable pragmatic experience in using this technology, focusing on femoro-popliteal disease.

## Methods

### Aim and Objectives

#### Main aim

The primary aim of this process was to achieve consensus regarding the best standards of care when using Shockwave IVL in steno-occlusive femoro-popliteal atherosclerotic lesions as part of an endovascular treatment for patients with symptomatic peripheral arterial disease (PAD).

### Objectives

1. Perform a scoping literature review/search to identify studies reporting on the use of Shockwave IVL in femoro-popliteal lesions and to inform subsequent surveying and the consensus process.
2. Identify expert stakeholders/participants to take part in the process, across Europe (and the United Kingdom).
3. Perform a structured online survey of participants.
4. Conduct an online event to discuss findings of the structured online survey and subsequent analysis of individuals’ opinions based on a modified Delphi approach.
5. Produce a list of themes and consensus areas to be approved by the expert group.

#### Approvals and regulatory considerations

This project did not involve patients, or anyone receiving medical/invasive treatment as part of this process. All participants were qualified health care professionals, recruited across Europe and the United Kingdom (see Appendix). Written informed consent was sought from all participants taking part in all relevant activities, either remote (online) or face-to-face. No identifiable data were recorded or shared at any point. All participants agreed to the analysis and recording of their views (written consent). The ANONYMISED FOR REVIEW provided confirmation that this project does not require ethical approval from an ANONYMISED FOR REVIEW Committee as it does not involve patients or use of patients’ data/information. The project was registered prospectively as a quality improvement project with ANONYMISED FOR REVIEW and also received ANONYMISED FOR REVIEW approval as a project not involving human subjects/patients (reference: ANONYMISED FOR REVIEW). No payments were made to those taking part in the project. The process was funded by Shockwave Medical Ltd, who covered teleconferencing costs, but did not take part in data analyses; raw data were not shared with the funder (no funding reference available).

### Process to Achieve Consensus and Finalize Recommendations of Best Practice

A 3-round online and face-to-face modified Delphi process^9^ was performed adhering to recommendations by COMET;^9–11^ standards and recommendations were split into 6 domains/themes reflecting the key areas of interest in this clinical context (informed by stage 1). We opted for the use of a modified version of the Delphi process, while still adhering to COMET guidance, to ensure that all participants had sufficient experience using the device/technology, as per the participant inclusion criteria listed below:

1. Preoperative assessment of lesion using cross-sectional imaging (ie, computed tomographic arteriography (CTA), magnetic resonance arteriography, ultrasonography, or other).
2. Intraoperative lesion imaging and assessment.
3. Rationale for use of Shockwave IVL.
4. Technical aspects of use of Shockwave IVL during the procedure.
5. Assessment of lesion following IVL use.
6. Decision-making regarding adjunctive treatment following use of IVL.

The 3-step modified Delphi process involved (in a stepwise manner):

1. Scoping literature review to inform the subsequent survey questions and focus-group discussion.
2. Structured online survey to explore key areas of interest.
3. Online focus group of expert stakeholders to address areas where further discussion was necessary in order to reach consensus. Further interviewing took place (one-to-one) with specific stakeholders to reach saturation regarding all topics.

The above provided the necessary qualitative and quantitative data which were then used to reach consensus based on the modified Delphi approach and complimentary qualitative analysis of both free text (survey) entries and views expressed in the focus group as well as subsequent interviews (to reach saturation).

### Scoping Review (First Step)

We searched the MEDLINE, EMBASE, AND SCOPUS databases to identify relevant literature regarding the use of IVL in femoro-popliteal lesions amongst patients with PAD (MeSH terms: peripheral arterial disease, lithotripsy). The search was performed by A.S. and N.T. as well as the lead University of Leicester Librarian using the Scopus literature search tools; 2 experts (A.S. and N.T.) discussed the identified titles, abstracts, and manuscripts (November 2023). A total of 7 articles were noted to be of interest and relevant for the subsequent online survey and were used in order to generate the questions and workshop/interview topic guides.^2–4,12–15^

### Online Structured Survey (Second Step)

An online structured survey was then designed by the lead authors (A.S. and N.T.) and S.J.M., based on the scoping review and considerations regarding preoperative/intraoperative/postoperative use of IVL in this clinical context, based on a discussion between A.S. and N.T. The survey used both closed questioning and free text entries; the survey was completed in the last quarter of 2023.

Participants were asked to rank the importance of each query explored in the survey (identified in the first step) using a Likert scale, as recommended by the Grading of Recommendations, Assessment, Development and Evaluation working group. Participants ranked items and differences in rankings between stakeholders were explored to finalize potential topics of discussion during the subsequent online event and eventually finalize the recommendations.

### Expert Stakeholders’ Event (Third Step)

Following completion of the survey, a stakeholders’ event (focus group) was hosted online in January 2024, to discuss views/opinions and areas where consensus could not be achieved based on the survey findings.

The event was hosted online and involved 15 participants; A.S. and N.T. led and facilitated the event.

### Inclusion Criteria for the Stakeholders’ Event

Inclusion criteria were as follows: adults over 18 years of age, who already provide health care for patients with PAD; previous experience treating steno-occlusive femoro-popliteal PAD using Shockwave IVL, having performed a minimum of 50 such procedures as a lead operator in the last 2 years; and able to understand, interpret, and communicate in English

The event was publicized on social media for 4 weeks (December 2023) and participants were also invited by the authors across Europe via e-mail. Participants were selected once replies were received, ensuring that all participants met the inclusion criteria listed above. A total of 51 participants replied and/or contacted the team, of which 1 eventually did not take part (no reason given).

A presentation by N.T. and A.S. was used to give structure to the event and 4 cases were also presented in order to visually discuss details surrounding areas of uncertainty. The presentation included introductions, definitions, and explanation of the study aims as well as IVL.

### Definitions

The femoro-popliteal segment for the purpose of this work was defined as any arterial segment between the bifurcation of the common femoral artery and the bifurcation of the popliteal artery (ie, above the crural arteries). The common femoral artery, profunda femoris, and external iliac artery are not covered by this work. All other definitions were as per the latest global guidelines on CLTI.^16^

### Analysis

The online survey results were analyzed in tabular format and key areas of interest were then discussed in the online event: preoperative, intraoperative, and postoperative considerations, surrounding clinical and technical decision-making. We also held further 4 face-to-face interviews with 5 participants to ensure that we reached saturation (as assessed by the lead authors and study group) and final consensus.

Comprehensive field notes were taken by the team during the online event and interviews. Thematic analysis was used to organize the views expressed in the interviews and the free text replies of the survey into themes.^17^ Those in attendance were invited to leave their email address to be contacted for comments they did not wish to share openly (2 participants shared further views with the lead author). No identifiable data (eg, date of birth or address) were collected; email addresses were stored in a hard disk drive for future contact (with the participants’ consent).

The shortlist of recommendations was reviewed by the core study group (N.T. and A.S.) to agree on the wording of the final set of standards for publishing, as recommended by the COMET Initiative and was also shared with all stakeholders prior to publication. These were then shared with all stakeholders completing the survey and taking part in the focus group for final feedback and subsequent consensus to be reached.

To reduce bias, a predetermined consensus threshold was used: standards ranked of critical importance^7–9^ by >70% or of little importance^1–3^ by <15% by participants were deemed to have reached the threshold for consensus for inclusion. Any items/recommendations that were ranked of critical importance^7–9^ by <10% of stakeholders or of little importance^1–3^ by >60% of the group were excluded from the final list of consensus points, following the online survey (first round) and online event (second round). In terms of agreement with regard to the subsequent phrasing of the recommendations, unanimous agreement was stated when all participants agreed with regard to a statement, high-level agreement was reached when ≥70% agreed and moderate agreement when 50% to 70% had agreed; of note, all statements described in this document reached at least 70% or higher agreement.

## Results

### Scoping Review

In the scoping review, we identified no universally-agreed consensus criteria regarding vessel preparation in this instance or clinical context, and no consensus recommendations or guidance regarding the use of lithotripsy in people with PAD. We identified 1 completed randomized trial (efficacy-driven) relating to PAD and use lithotripsy, which was used to inform our survey (PAD 3 trial).^15^

### Survey and Online Event

The survey was completed in full by 25 stakeholders (Appendix), from 6 European countries in the last quarter of 2023 (Appendix). All those invited filled in the survey with no missing entries and all provided free text answers. The subsequent online event attended by 15 stakeholders, with an additional 9 interviews (online—one-to-one) conducted for those who could not attend, to ensure data saturation at the last stage of the modified consensus process.

The Appendix details characteristics of the 25 stakeholders who took part in the survey and online event/interviews to finalize areas of disagreement or discrepancies in the survey(s). Of note, most (16; 62%) were vascular surgeons and the rest were interventional radiologists/cardiologists with an additional 2 angiologists (who also perform vascular procedures). The geographical reach of the collaborators taking is detailed in the Appendix and covered a considerable part of Europe/United Kingdom. All participants were independent practitioners (equivalent to consultant physicians/doctors) and were working as part of a vascular unit/department in a teaching hospital.

### List of Consensus Recommendations

#### Preoperative assessment of lesion using cross-sectional imaging (ie, computed tomographic arteriography, magnetic resonance arteriography, ultrasonography, or other)

Patients considered for endovascular treatment of the femoro-popliteal segment with the potential for use of Shockwave IVL, should undergo imaging assessment with cross-sectional means including at least a CTA and duplex ultrasonographic assessment of the femoro-popliteal lesions in question (Table 1).

**Table 1.** Recommendations When Using Shockwave Intravascular Lithotripsy (IVL) in Femoro-Popliteal Lesions.

Area of consensus and recommendation	Agreement reached across 3 Delphi cycles
Preoperative assessment of any target lesion using cross-sectional imaging (ie, computed tomographic arteriography, magnetic resonance arteriography, ultrasonography, or other) should be performed in all patients where Shockwave IVL is considered as a treatment option.	100%
The characteristics of the calcium load of the plaque/lesion should be verified intraoperatively, ideally using at least 2 views with digital subtraction angiography. If intravascular ultrasound is available, that could be employed to further characterize calcium characteristics and length of the plaque; however, that is not required in all cases.	92%
Three-dimensional reconstruction of the CTA should be used with center-line assessment in order to identify calcified plaque elements and exact vessel diameters, alongside the preoperative duplex.	100%
The assessment of calcium load within a plaque should be based on a combination of the above techniques (CTA, ultrasonography, angiography), using the operator’s preferred mode of quantifying calcium.	100%
A diagnostic angiogram might be required in case of non-agreement regarding the nature and size of the lesion in question, but is not routinely required.	100%
The Peripheral Arterial Calcium Scoring System (PACSS) should be used when quantifying calcium load,^5,6,18^ but such a quantification is not necessarily always required outside a research setting.	90%
Intraoperative lesion imaging and assessment, including use of intravascular ultrasound (IVUS) and lesion sizing	
All patients should undergo assessment of the target femoro-popliteal lesion with at least 2 separate views of the lesion using angiography, at a different angle, in order to assess the lesion in question in at least 2 different planes, which will allow better understanding of residual disease (following initial treatment), calcium modification impact, and potential dissection(s).	100%
If intravascular ultrasound (IVUS) is available, that could be employed to further characterize calcium characteristics and length of the plaque; however, that is not required to use the device. The latter is more useful in lesions where preoperative imaging might have identified a potential thrombotic element of the lesion in question and could be used to verify such preoperative cross-sectional findings.	80%
Extravascular ultrasound (EVUS) capabilities should be available in the operating suite when performing a femoro-popliteal procedure of this nature; however, the use of EVUS should be left down to the operator to decide.	72%
Target vessel diameter at the lesion location should be calculated based on cross-sectional preoperative imaging (CTA and ultrasonography) at the widest part of the calcified lesion (outer to outer).	100%
Technical aspects of use of Shockwave IVL during the procedure	
An ipsilateral femoral approach should be used, unless not indicated.	100%
An intraluminal-first crossing strategy with appropriate wires should be attempted in all cases. The panel found that .014 or .018 wires should be used where possible; however, these technical considerations should be left to each operator to decide.	92%
At least 2 cycles of Shockwave IVL therapy should be applied in all lesions.	100%
Instructions for use should be followed in terms of preparing the device (Shockwave catheter) and subsequent procedural steps; however, oversizing and inflation at lower atmospheric pressure can be used by the operators as per their preferences and lesion characteristics.	100%
For eccentric or coralline lesions, oversizing should be applied (with regard to choice of Shockwave IVL catheter size) to ensure the whole lesion is covered by the relevant catheter. This might require the use of multiple catheters. The oversizing should be by at least 1 mm of the diameter of the artery at the level of the eccentric/coralline target lesion.	100%
The lesion should be treated from healthy-to-healthy artery, based on preoperative and intraoperative lesion length assessment.	100%
Crossing the lesion in a subintimal plane is not prohibitive regarding the subsequent use of Shockwave IVL, but the application of energy to the plaque might be less efficient, and therefore, assessment of lumen formation with ultrasound might be necessary (moderate agreement).	92%
Shockwave IVL should not be used as a vessel preparation stand-alone therapy in embolic or recently thrombotic (≤2 weeks) lesions. The assessment of the lesion’s consistency should take place preoperatively (with regard to embolic/thrombotic burden).	100%
In case of inability to cross a tightly stenosed calcified lesion or chronic total occlusion (CTO), the operator(s) can use a 3 mm or smaller balloon to predilate and allow catheter crossing	Confirmed via additional (post hoc) communication with 100% of participants.
The use of a peripheral embolic protection device is not necessary in all cases, unless particular lesion or patient-related characteristics necessitate the use of such a device.	100%
Assessment of lesion following Shockwave IVL application	
All procedures should be performed in a suite or operating theater equipped with angiographic capabilities, allowing the acquisition of imaging in at least 2 different planes, and with ultrasonographic capabilities on site (in the same suite), by operators trained in the procedure.	100%
The use of IVUS is not mandatory in all lesions following application of Shockwave IVL but may help to provide more information in lesions, such as accurate lumen gain, visualization of calcium modification (eg, cracks), and presence of significant dissection(s), which may not be detectable via angiography.	100%
The use of EVUS is highly recommended in longer lesions (moderate agreement), lesions of more than 5 mm in diameter, and especially those ≥20 cm, PACSS 3 and above lesions, or in case of sizing uncertainty or in case of a potential dissection.	88%
EVUS should be used in all cases if gaining percutaneous access to an artery for the subsequent Shockwave IVL procedure.	100%
All lesions should be assessed using angiography in at least 2 views (unanimous agreement) as a bare minimum, before and following the use of lithotripsy.	100%
In wider arteries (≥5.5 mm) angiographic luminal gain in isolation is not the only indicator of procedural Shockwave IVL success, and the use of EVUS (or other imaging modality such as IVUS) is strongly recommended.	100%
Decision-making regarding adjunctive treatment following use of Shockwave IVL	
At least plain balloon angioplasty is necessary following the application of Shockwave IVL (based on preoperative and intraoperative imaging in terms of sizing the arterial diameter).	76%
Drug-coated balloon angioplasty should be used in PACSS 3 and above lesions following Shockwave IVL, unless stenting is required (and decided by the operator); however, the use of specific devices and drugs is left to the operator to decide, based on local preferences.	100%
Flow-limiting dissections require stenting, the choice of which should be left at the discretion of the operator.	100%
In a subintimal plane, the use of subsequent stenting might be more likely to be required, but should be left at the discretion of the operator, based on intraoperative imaging, with the use of 2-plane angiography deemed (unanimously) as necessary post-IVL use in this scenario.	76%
Atherectomy is not required routinely before or following Shockwave IVL.	92%
A completion angiogram at least 5 minutes after the application of lithotripsy and subsequent treatment (eg, a stent) should be performed to ensure that there is no recoil, given the nature of the calcified plaques in question.	96%
Duplex ultrasonography surveillance should be used for calcified lesions treated with IVL, with a duplex performed ideally at 30 days post-procedurally and subsequent follow-up and/or reintervention strategy left to the operators’ discretion, given the lack of high-quality randomized data in this domain.	95%
Post-procedural best medical therapy should include antithrombotic treatment based on latest available guidance.^19^	100%

The assessment of calcium load should be based on a combination of the above techniques, using the operator’s preferred mode of quantifying calcium. Three-dimensional reconstruction of the CTA should be used with center-line assessment in order to identify calcified plaque elements and exact vessel diameters, alongside the preoperative duplex.

A diagnostic angiogram might be required in case of non-agreement regarding the nature and size of the lesion in question, but is not routinely required. The Peripheral Arterial Calcium Scoring System (PACSS) was deemed as the most relevant scoring system to be used in this instance in terms of quantifying calcium load,^5,6,18^ but such a quantification is not necessarily required outside a research setting.

The characteristics of the calcium load of the plaque should then be verified intraoperatively, ideally using at least 2 views with digital subtraction angiography. If intravascular ultrasound (IVUS) is available, that could be employed to further characterize calcium characteristics and length of the plaque; however, that is not required in all cases.

#### Intraoperative lesion imaging and assessment, including use of intravascular ultrasound

All patients should undergo assessment of the target femoro-popliteal lesion with at least 2 separate views of the lesion using angiography, at a different angle, in order to assess the lesion in question in at least 2 different planes, which will allow better understanding of residual disease (following initial treatment), calcium modification impact, and potential dissection(s). If IVUS is available, that could be employed to further characterize calcium characteristics and length of the plaque; however, that is not required to use the device. The latter is more useful in lesions where preoperative imaging might have identified a potential thrombotic element of the lesion in question and could be used to verify such preoperative cross-sectional findings.

Extravascular ultrasound (EVUS) was unanimously found to be useful in terms of characterizing a calcified lesion intraoperatively as well as a tool to verify the success of the revascularization attempt. A third of participants found that EVUS also allows more precise diameter measurements and length of lesion determination.^20^ Extravascular ultrasound capabilities should be available in the operating suite when performing a femoro-popliteal procedure of this nature; however, the use of EVUS should be left down to the operator to decide.^20^

Target vessel diameter at the lesion location should be calculated based on cross-sectional preoperative imaging (CTA and ultrasonography) at the widest part of the calcified lesion (outer to outer); highest level of agreement.

#### Rationale for use of Shockwave intravascular lithotripsy

Shockwave IVL in the femoro-popliteal segment is best suited (ideal best-case scenario) in the case of circumferential severely calcified lesions of less than 20 cm in length and when the crossing of the lesion has taken place intraluminally (highest level of agreement).

At the same time, the exact length of the lesion is not necessarily a criterion for the use or non-use of IVL, and longer lesions might be treated efficiently, but this may require the use of more than 1 catheter.

Shockwave IVL can also be used efficiently in eccentric lesions or “coralline” femoro-popliteal lesions; however, more cycles of treatment will have to be applied for adequate calcium modification.

There was high-level agreement that an intraluminal-first strategy should be followed whenever planning to use IVL in the femoro-popliteal segment; however, the technology can be used safely in the subintimal space. In the case of the latter, there was moderate level of agreement that ultrasound-based imaging adjuncts (intravascular/extravascular) should be used to assess the effect of lithotripsy.

#### Technical aspects of use of Shockwave intravascular lithotripsy during the procedure

There was the highest level of agreement that, where possible, an ipsilateral femoral approach should be used, unless not indicated.

There was unanimous agreement that an intraluminal-first crossing strategy with appropriate wires should be attempted in all cases. The panel found that .014 or .018 wires should be used where possible; however, these technical considerations should be left to each operator to decide. There was unanimous agreement that at least 2 cycles of Shockwave IVL therapy should be applied in all lesions.

There was unanimous agreement that instructions for use should be followed in terms of preparing the device (Shockwave catheter) and subsequent procedural steps; however, there was high-level agreement that oversizing and inflation at lower atmospheric pressure can be used by the operators as per their preferences and lesion characteristics.

For eccentric or coralline lesions, there was unanimous agreement that oversizing should be applied (with regard to choice of Shockwave catheter size) to ensure the whole lesion is covered by the relevant catheter. This might require the use of multiple catheters. The oversizing should be by at least 1 mm of the diameter of the artery at the level of the eccentric/coralline target lesion. Overall, operators felt confident significantly oversizing the device based on procedural experience. The lesion diameter should be sized based on preoperative cross-sectional imaging (CTA and ultrasonography), confirmed intraoperatively via at least 2 angiographic views (as a minimum).

The lesion should be treated from healthy-to-healthy artery, based on preoperative and intraoperative lesion length assessment. Crossing the lesion in a subintimal plane is not prohibitive regarding the subsequent use of Shockwave IVL, but the application of energy to the plaque might be less efficient, and therefore, assessment of lumen formation with ultrasound might be necessary (moderate agreement).

Shockwave IVL should not be used as a vessel preparation stand-alone therapy in embolic or recently thrombotic (≤2 weeks) lesions. The assessment of the lesion’s consistency should take place preoperatively (with regard to embolic/thrombotic burden).

There was unanimous agreement that the use of a peripheral embolic protection device is not necessary in all cases, unless particular lesion or patient-related characteristics necessitate the use of such a device.

#### Assessment of lesion following intravascular lithotripsy use

All procedures should be performed in a suite or operating theater equipped with angiographic capabilities, allowing the acquisition of imaging in at least 2 different planes, and with ultrasonographic capabilities on site (in the same suite), by operators trained in the procedure.

The use of IVUS is not mandatory in all lesions following application of Shockwave IVL but may help to provide more information in lesions, such as accurate lumen gain, visualization of calcium modification (eg, cracks), and presence of significant dissection(s), which may not be detectable via angiography. The use of EVUS was recommended in longer lesions (moderate agreement), lesions of more than 5 mm in diameter, and especially those ≥20 cm, PACSS 3 and above lesions, or in case of sizing uncertainty or in case of a potential dissection.

Extravascular ultrasound should be used in all cases if gaining percutaneous access to an artery for the subsequent Shockwave IVL procedure. All lesions should be assessed using angiography in at least 2 views (unanimous agreement) as a bare minimum, before and following the use of lithotripsy.

In wider arteries (≥5.5 mm) angiographic luminal gain in isolation is not the only indicator of procedural Shockwave IVL success, and the use of EVUS (or other imaging modality such as IVUS) is strongly recommended.

#### Decision-making regarding adjunctive treatment following use of Shockwave intravascular lithotripsy

There was moderate-level agreement that at least plain balloon angioplasty is necessary following the application of Shockwave IVL (based on preoperative and intraoperative imaging in terms of sizing the arterial diameter).

Drug-coated balloon angioplasty should be used in PACSS 3 and above lesions following Shockwave IVL, unless stenting is required (and decided by the operator); however, the use of specific devices and drugs is left to the operator to decide, based on local preferences.

There was unanimous agreement that flow-limiting dissections require stenting, the choice of which should be left at the discretion of the operator. There was no agreement regarding type of stenting as this should be best left to the operator and/or clinical team to decide based on local practice(s) and availability of relevant devices. There was moderate level of agreement that in a subintimal plane, the use of subsequent stenting might be more likely to be required, but should be left at the discretion of the operator, based on intra-operative imaging, with the use of 2-plane angiography deemed (unanimously) as necessary post-IVL use in this scenario.

There was unanimous agreement that atherectomy is not required routinely before or following Shockwave IVL. There was high-level agreement that a completion angiogram at least 5 minutes after the application of lithotripsy, and subsequent therapy should be performed to ensure that there is no recoil, given the nature of the calcified plaques in question.

There was high-level agreement that duplex ultrasonography surveillance should be used for these lesions, with a duplex performed ideally at 30 days postprocedurally and subsequent follow-up and/or reintervention strategy left to the operators’ discretion, given the lack of high-quality randomized data in this domain.

There was high-level agreement that postprocedural best medical therapy should include antithrombotic treatment based on latest available guidance.^19^

## Discussion

Shockwave IVL has been introduced as a helpful tool in endovascular peripheral practice in the last few years, following its adoption in coronary disease minimally invasive treatment.^1–4,12–14^

Previously, within the PAD 3 randomized controlled trial, an efficacy-driven Shockwave IVL study in PAD, IVL was shown to be superior to plain percutaneous transluminal angioplasty (PTA) in terms of procedural success and short-term complications relating to the lesion. Vessel preparation with IVL was safely performed using a significantly lower maximum inflation pressure relative to PTA, resulting in lower rates of dissections and a lower risk of postdilatation and provisional stent placement. The investigators concluded that IVL may be a valuable tool for treating patients with calcified arterial lesions, potentially leading to improved outcomes and reduced complications in the management of PAD with heavily calcified arteries.

In a real-world setting, the use of peripheral IVL has demonstrated low residual stenosis, high acute luminal gain, and a low rate of complications despite the complexity of the disease typically treated with this type of technology, ie, severely calcified arteries.^2,12^ In PAD 3 and the associated cohort study (all-comers treated with IVL), there was an average acute gain of 3.4 mm at the end of the procedure and a final mean residual stenosis of 23.6%. In addition, angiographic complications were rare, with a single perforation following drug-coated balloon inflation, unrelated of the IVL procedure out of the 114 femoro-popliteal lesions analyzed in the observational real-world cohort study performed alongside of the PAD 3.

The above literature has led to a wider adoption of Shockwave IVL in PAD endovascular practice. Given the high complexity and calcific load/burden of these lesions, it is logical to expect that operators’ practice and views with regard to how IVL should be used in this instance would vary. This project aimed to address this issue and provide clarity as to how this technology should be used in a best-case scenario as well as typical routine care. The expert team involved focused on areas of uncertainty, following a scoping review, preoperatively/intraoperatively/postoperatively. The subsequent recommendations have been made based on a stringent stepwise approach in order to achieve high-level consensus on all statements produced by the group. The recommendations by this group of experts do not replace the instructions for use of Shockwave IVL. It acts as a complementary tool in order to aid clinicians/operators in daily decision-making. The team focused on areas of particular uncertainty and areas where disagreement is common among operators. The subsequent recommendations are a high-quality guide to best practice in this clinical context and can be used for those undergoing PAD intervention with femoro-popliteal disease.

One key area to note it the use of a small balloon to predilate a long calcified lesion prior to advancing an IVL catheter; these was mentioned by some participants as a useful adjunct (using a 3 mm balloon to predilate the lesion in question), but was not brought up as a recommendation relevant for inclusion in this report during the formal Delphi process. It is, however, a commonly performed technical maneuver and the group agreed that it is safe for tightly stenosis lesions as well as chronic total occlusions. The group agreed to include this as a potential useful maneuver for such lesions.

## Limitations

This work was funded by Shockwave; however, the funder did not analyze data and did not interfere in the consensus design process. Results were analyzed by the authors who prepared this manuscript/report. The participants were chosen based on the criteria described in this article; they mostly cover European regions and this might have impacted on subsequent recommendations. It is impossible, however, to involve operators from all areas/regions where IVL is used and the international nature of this consensus group means that the recommendations are applicable across many European health care providers. With regard to calcium scoring methods and scores, this panel preferred the PACSS score; however, the DISRUPT PAD trials have utilized the Peripheral Academic Research Consortium (PARC) score. The panel did not specifically comment on the use of the PARC score. The use of IVL within stents for stenoses or occlusions was not commented upon by the panel as this is not recommended by the manufacturer. Finally, the panel did not make any recommendations about the use of predilation in order to advance IVL catheters in long lesions.

## Conclusion

This report provides an aid for operators using Shockwave IVL to treat femoro-popliteal PAD, which should aid decision-making as an adjunct to the technical instructions for use provided by the manufacturer.

## Appendix

**Table:** List of Collaborators^a,b^ Who Took Part in the Consensus Process (Both the Survey and Online Work Shop +/- Interview).

Surname	First name	Profession	Country
Alejandre Lafont	Enrique	Vascular Surgeon	Spain
Bonvini	Stefano	Vascular Surgeon	Italy
Bosiers	Michel	Vascular Surgeon	Switzerland and Germany
Coscas	Raphael	Vascular Surgeon	France
Davies	Robert	Vascular Surgeon	The United Kingdom
Del Canto Peruyera	Pablo	Vascular Surgeon	Spain
Elias	Noory	Cardiologist and Angiologist	Germany
Fazzini	Stefano	Vascular Surgeon	Italy
Garriboli	Luca	Vascular Surgeon	Italy
Goyault	Gilles	Vascular Surgeon	France
Howard	Dominic PJ	Vascular Surgeon	The United Kingdom
Huasen	Bella	Interventional Radiologist	The United Kingdom
Isernia	Giacomo	Vascular Surgeon	Italy
Lakshminarayan	Raghu	Interventional Radiologist	The United Kingdom
Patel	Ashish	Vascular Surgeon	The United Kingdom
Patel	Rafiuddin	Interventional Radiologist	The United Kingdom
Patrone	Lorenzo	Interventional Radiologist	The United Kingdom and Italy
Portou	Mark	Vascular Surgeon	The United Kingdom
Rammos	Christos	Cardiologist	Germany
Ruffino	Maria Antonella	Interventional Radiologist	Switzerland
Sirvent	Marc	Vascular Surgeon	Spain
Sritharan	Kaji	Vascular Surgeon	The United Kingdom
Vijaynagar	Badri	Vascular Surgeon	The United Kingdom
von Stempel	Conrad	Interventional Radiologist	The United Kingdom
Zanabili	Amer	Vascular Surgeon	Spain

a All the above collaborators have reviewed, edited, and approved this report.

b The authors’ group (Saratzis, Messeder, Narayanan) are based in the United Kingdom.
